# New combined surgery for cervical cancer complicated by pelvic organ prolapse using autologous fascia lata: A case report

**DOI:** 10.1002/ccr3.2883

**Published:** 2020-05-20

**Authors:** Yukihide Ota, Yukio Suzuki, Tatsuya Matsunaga, Ryunosuke Ninomiya, Shintaro Kagimoto, Etsuko Miyagi

**Affiliations:** ^1^ Department of Obstetrics and Gynecology Yokohama City University Graduate School of Medicine Yokohama Japan; ^2^ Department of Plastic Surgery Yokohama City University Graduate School of Medicine Yokohama Japan

**Keywords:** autologous fascia lata, cervical cancer, incontinence, pelvic organ prolapse, sacral colpopexy

## Abstract

Radical hysterectomy and immediate sacral colpopexy using autologous fascia lata could be considered a treatment option for cervical cancer complicated by severe and symptomatic pelvic organ prolapse.

## Graphical abstract

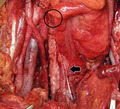

## 1 INTRODUCTION

Cervical cancer rarely coexists with pelvic organ prolapse. We report the first case of radical hysterectomy and immediate sacral colpopexy using autologous fascia lata. This combined surgery is feasible and safe treatment options for early‐stage cervical cancer complicated by severe and symptomatic pelvic organ prolapse.

Cervical cancer and pelvic organ prolapse (POP) are both common diseases. However, cervical cancer as a comorbid disease in those with POP is extremely rare, with an incidence of only 0.14% to 1%.^1^ Thus, no standard treatment modality has been established for this comorbid condition.

Both radical surgery and radiotherapy yield good outcomes in early‐stage cervical cancer, with a 5‐year overall survival rate of 83% for either treatment and 20‐year overall survival rate of 72% for radical surgery and 77% for radiotherapy.^2^, ^3^ Although radiotherapy and concurrent chemoradiotherapy may be among the optimal options for curative treatment, a previous study reported that surgery may have a positive effect on overall survival for stage I to IV cervical cancer complicated by POP.^1^

In general, there are many treatment modalities available for POP, namely pessary, colpocleisis, colporrhaphy, sacrospinous colpopexy, transvaginal mesh, and sacral colpopexy. Pessary is an easy method; however, it is not suitable for severe POP. Colpocleisis, though a minimally invasive surgery, does not allow for easy follow‐up of patients since the vagina is closed as part of the procedure precluding vaginal smear assessments. Sacral colpopexy has a lower recurrence rate for POP than colporrhaphy, sacrospinous colpopexy, and transvaginal mesh.^4^ A synthetic mesh is usually used for sacral colpopexy and transvaginal mesh, which can reportedly lead to vaginal erosion in 3.2%‐12% of cases.^5^

Here, we confirm that the newly devised technique for treating both cervical cancer and POP‐related symptoms is safe and feasible. Furthermore, we report a case of locally advanced cervical cancer complicated by symptomatic POP that was treated with the new surgical technique.

### 1.1 Questionnaires

There are three main questionnaires available for subjective assessment of POP, namely Core Lower Urinary Tract Symptom Score (CLSS), Overactive Bladder Symptoms Score (OABSS), and King's Health Questionnaire (KHQ). CLSS provides a comprehensive assessment of 10 lower urinary tract symptoms (LUTSs) (Table S1).^6^ OABSS evaluates and quantifies four symptoms of overactive bladder (Table S2).^7^ KHQ is a validated and reliable scale for the assessment of quality of life (QOL) in women with urinary incontinence.^8^ It can be accessed via The Permanente Medical Group website.^9^ In all three self‐report questionnaires, a lower score indicates better QOL.

## 2 CASE REPORT

**Figure 1:**
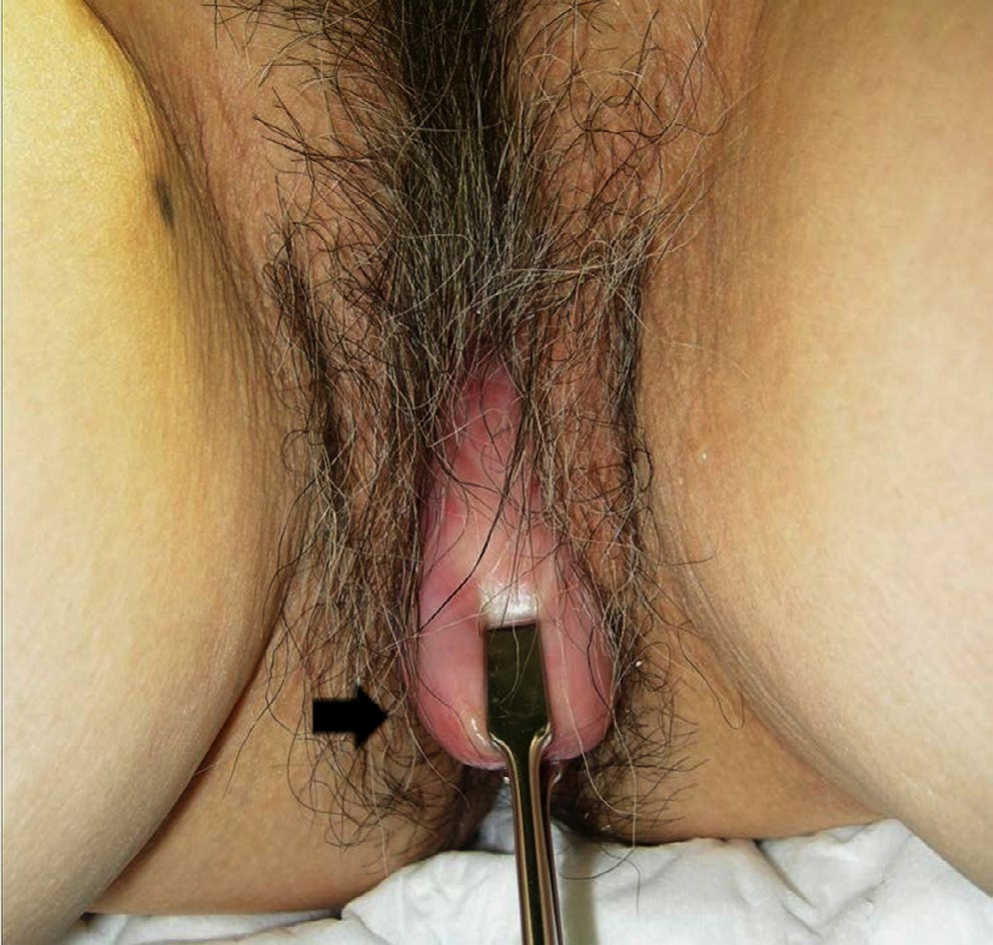
Visual examination for cervix. Cervix can be seen 3 cm below the vaginal orifice (black arrow)

A 71‐year‐old Japanese woman, gravida 3, para 3, presented to our institution complaining of genital bleeding, with frequent and difficult urination. She had no remarkable past medical or family history of malignancy or illness. Physical examination revealed POP (Pelvic Organ Prolapse Quantification stage III) (Figure 1, Table 1) with a 3‐cm‐sized ulcerated lesion on the cervix. Colposcopy showed atypical vessels and an ulcerated lesion on the entire cervical circumference suggestive of invasive carcinoma. Biopsy of the cervix confirmed squamous cell carcinoma (SCC), clinical stage IB1 (The International Federation of Gynecology and Obstetrics) and T1b1N0M0. The treatment of choice for this stage of cervical cancer in our hospital is abdominal radical hysterectomy, radiotherapy, or concurrent chemoradiotherapy as stipulated in The Japanese Society of Gynecologic Oncology 2017 guidelines on cervical cancer treatment.^10^ Our patient was expected to have a good long‐term prognosis after surgical intervention. Hence, the main challenge was to improve QOL by addressing the POP. We were not convinced that the use of synthetic mesh would be the best approach for POP in this case, as we suspected that the mesh might be influenced by cancer recurrence, chemotherapy, and radiotherapy or affect the treatment procedure or the recurrence of the disease. Hence, we used autologous fascia lata as a substitute for synthetic mesh and performed abdominal radical hysterectomy and immediate sacral colpopexy using an autologous fascia lata graft to simultaneously treat cervical cancer and POP.

**Table 1:** POP‐Q system and Self‐reported questionnaires

			Preoperative			3 mo postoperative			20 mo postoperative		
POP‐Q^a^ system			stage III			stage 0			stage 0		
Aa	Ba	C	0	+3	+3	−3	−3	−5	−2	−3	−5
gh	pb	tvl	3	1	7	3	1	5	3	1	5
Ap	Bp	D	0	0	+3	−3	−3	N/A^b^	−3	−3	N/A
CLSS^c^			11			7			7		
OABSS^d^			6			3			2		
KHQ^e^			193			191			131		

Note

The POP‐Q system shows remarkable anatomical improvement in the anterior (Aa and Ba) and posterior (Ap and Bp) vaginal prolapses. The cervix has been modified into a vaginal cuff and fixed on a higher position. All three scores decreased and show the improvement in the subjective symptoms.

^a^ Pelvic Organ Prolapse Quantification.

^b^ Not applicable.

^c^ Core Lower Urinary Tract Symptom Score.

^d^ Overactive Bladder Symptoms Score.

^e^ King's Health Questionnaire.

**Figure 2:**
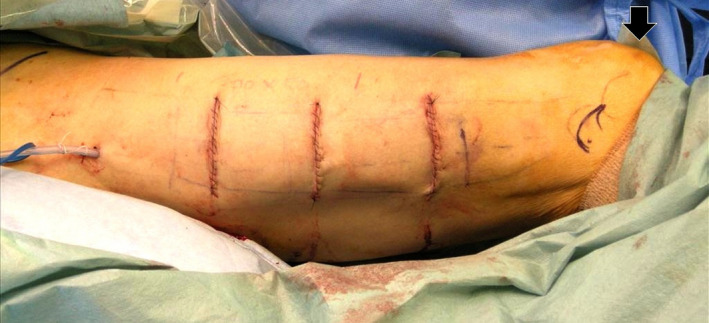
The donor site (right thigh). Three 6‐cm linear incisions were made on the right lateral/anterior thigh. Solid arrow indicates the right patella

**Figure 3:**
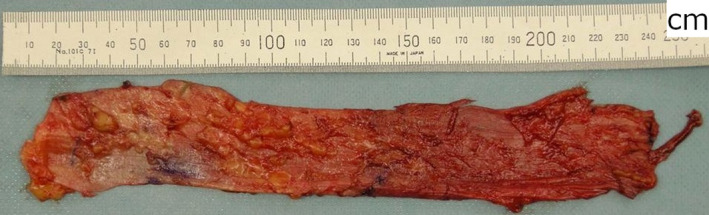
The harvested graft. The obtained anterior fascia lata sheet (20 × 5 cm)

**Figure 4:**
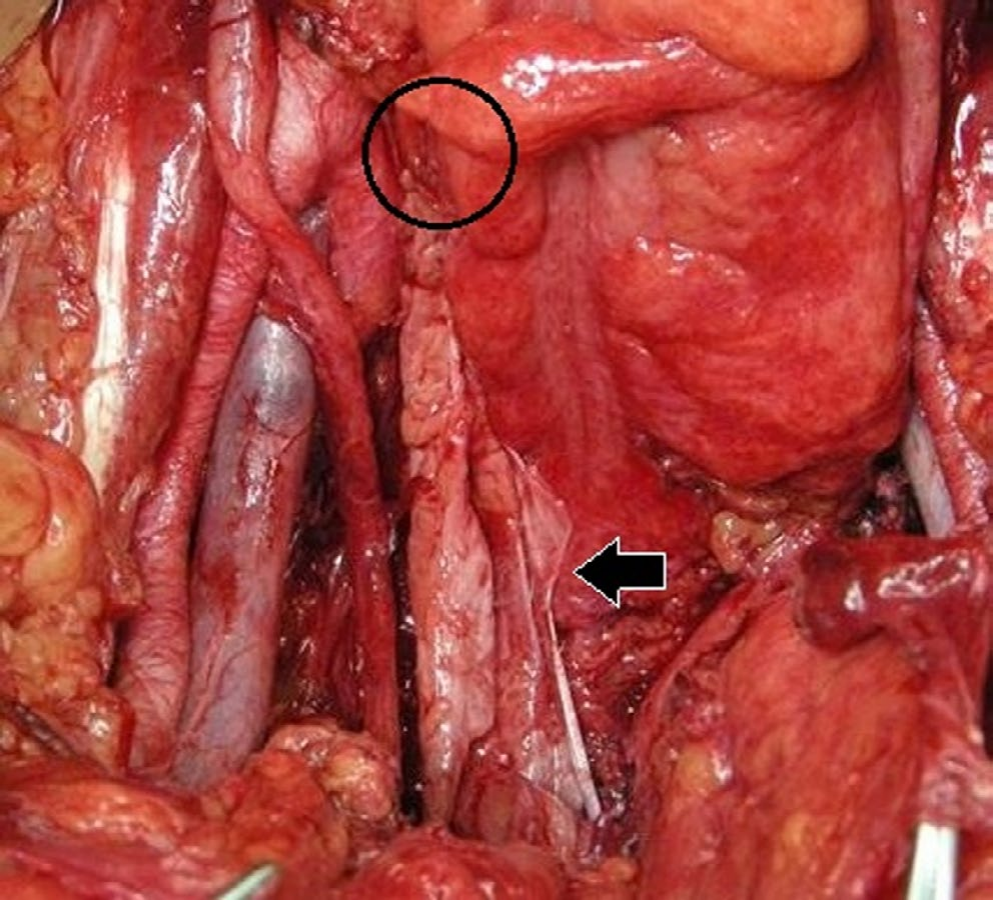
Intraoperative findings. The anterior fascia lata sheet was fixed to the anterior vaginal wall and sacral promontory. Solid arrow indicates anterior fascia lata. Promontory is marked with a circle

The patient underwent abdominal nerve‐sparing radical hysterectomy. Plastic surgeons then identified the distal and proximal attachments of the fascia lata. We measured the length of the fascia lata. The first 6‐cm linear incision was made at a point two‐thirds proximal to the fascia's distal attachment. Two more 6‐cm linear incisions were made 7 cm proximal and distal to the first incision (Figure 2). Subcutaneous fat was bluntly dissected, and a 20 cm × 5 cm fascia lata sheet was obtained (Figure 3). The distal aspect of the autologous fascia lata was fixed to the anterior vaginal wall, and the vaginal cuff was closed. The cephalad aspect of the autologous fascia lata was fixed to the anterior longitudinal ligament at the sacral promontory (Figure 4). We placed antiadhesion products on retroperitoneal defects and did not close the retroperitoneal defects. No severe complications occurred in either the abdomen or the donor site. She left the hospital on the 21st postoperative day. Microscopic examination of the surgical specimen showed nonkeratinizing SCC of the cervix without lymphovascular invasion and negative surgical margin. The pathological stage was pT1b1N0M0.

POP was anatomically improved after surgery (Table 1), and there was no sign of recurrence 20 months after surgery. According to the pelvic organ prolapse quantification system, the patient demonstrated elevation of Aa, Ba, and C points which contributed to improved urination. After treatment, the patient did not experience difficult or frequent urination. Alongside the surgical intervention, we used three self‐report questionnaires before and after surgery to evaluate QOL in the patient. The total scores of CLSS, OABSS, and KHQ had decreased, confirming improved QOL (Table 1). There were no signs of recurrence of cervical cancer 20 months after surgery.

All procedures in this case report were approved by Yokohama City University Hospital Institutional Ethics Review Board. The patient provided written informed consent prior to the surgery.

## 3 DISCUSSION/CONCLUSION

This is the first case of a radical hysterectomy and immediate sacral colpopexy with an autologous fascia lata (RISA) graft for cervical cancer complicated by POP. Although this is a new surgical strategy, it is in fact comprised of well‐practiced surgeries. Therefore, it would be a feasible novel surgical technique for patients with cervical cancer complicated by POP in terms of both curative treatment and QOL improvement.

Radical hysterectomy, radiotherapy, and concurrent chemoradiotherapy are the primary treatment options for early‐stage cervical cancer.^2^ Radiotherapy and concurrent chemoradiotherapy may be less invasive than radical hysterectomy and suitable for frail patients. In addition, subsequent surgery for POP is rarely performed because shrinkage of the uterus and supporting structures helps in repositioning of the pelvic organ.^11^ However, there is no evidence that LUTS‐related QOL improves after radiation. Moreover, radiotherapy and concurrent chemoradiotherapy have two disadvantages in cervical cancer complicated by POP. First, survival outcomes have been more favorable with surgery‐based treatment than with radiation‐based therapy for stages I to IV cervical cancer (5‐year recurrence‐free survival rate: 72.0% vs. 62.9%; 5‐year disease‐specific overall survival rate: 77.0% vs. 68.2%).^1^ Second, tumor regression and organ motion can affect the radiation dose,^12^ and cystocele‐rectocele may increase the risk of visceral radiation injury^13^ that lowers QOL.

Radical hysterectomy is a highly invasive surgery and may temporarily impair the function of the urinary system. In contrast, good long‐term prognosis and improvement of QOL can be expected via radical hysterectomy and immediate reconstruction surgery for POP. We selected sacral colpopexy because it has a lower recurrence rate for POP and is optimal for the Injury of Delancy^14^ levels 1 and 2. Abdominal sacral colpopexy was first reported by Ameline^15^ and Lane.^16^ Biological grafts were used to suspend the vaginal wall in both reports. Birnbaum^17^ was the first to describe the use of synthetic mesh in 1973. Since then, synthetic mesh has been used for sacral colpopexy. The objective failure rate for recurrence was significantly worse when using cadaveric fascial graft than when using synthetic mesh (32% vs. 9%; RR: 3.58).^4^ Some studies reported no vaginal erosion with an autologous graft.^18^ Sacral colpopexy should be performed immediately after radical hysterectomy because adhesiolysis in the second surgery may increase the risk of vaginal complications, including visceral injury and vaginal fistula, that can lower QOL. To minimize such surgical complications, we did not perform additional procedures designed to prevent occult urinary stress incontinence or anterior/posterior compartment prolapse after the procedure. RISA is feasible and effective for the simultaneous treatment of cervical cancer and POP in a single surgical procedure. In addition, RISA is applicable to patients with early‐stage cancer that can be completely removed and those who can endure highly invasive surgery. Gynecologists may not be familiar with the technique for harvesting the fascia lata sheet; however, plastic surgeons can perform it efficiently.^19^, ^20^

In conclusion, RISA is an effective treatment option for early‐stage cervical cancer complicated by severe and symptomatic POP. A larger number of reports on this topic should be assessed to help verify an optimal treatment modality for this disease.

## CONFLICT OF INTEREST

The authors have no conflicts of interest to declare. This study received no funding from public, private, or non‐for‐profit sectors.

## AUTHOR CONTRIBUTIONS

Yukihide Ota: conceived the study and contributed to carried out the experiment, wrote the manuscript in consultation with Yukio Suzuki. Yukio Suzuki, Tatsuya Matsunaga, Ryunosuke Ninomiya, Shintaro Kagimoto, and Etsuko Miyagi: contributed to experiments. Etsuko Miyagi was in charge of overall direction and planning. All authors provided critical feedback and helped shape the research, analysis, and manuscript.

## Supporting information

Table S1

Table S2
