# Supplementary material for: New combined surgery for cervical cancer complicated by pelvic organ prolapse using autologous fascia lata: A case report
Source: Clin Case Rep. 2020 May 20;8(8):1382–6. doi: 10.1002/ccr3.2883 (PMC7455420; doi:10.1002/ccr3.2883)
Supplement: Supplementary file 1 — Table S1 [file CCR3-8-1382-s001.docx]

Supplementary Table 1 - Core Lower Urinary Tract Symptom Score (CLSS) Questionnaire (modified from Homma Y et al^6^)

| *Please circle the number that applies best to your urinary condition during the last week.* | | | | | | |
| --- | --- | --- | --- | --- | --- | --- |
| How many times do you typically urinate from waking in the morning until sleeping at night? | *<7* | *8–9* | | *10–14* | | *>15* |
|  | 0 | 1 | | 2 | | 3 |
| How many times do you typically urinate from sleeping at night until waking in the morning? | *0* | *1* | | *2-3* | | *>4* |
|  | 0 | 1 | | 2 | | 3 |
| *How often do you have the following symptoms?* | *No* | *Rarely* | | *Sometimes* | | *Often* |
| A sudden strong desire to urinate, which is difﬁcult to postpone | 0 | 1 | | 2 | | 3 |
| Leaking of urine because you cannot hold it | 0 | 1 | | 2 | | 3 |
| Leaking of urine, when you cough, sneeze, or strain | 0 | 1 | | 2 | | 3 |
| Slow urinary stream | 0 | 1 | | 2 | | 3 |
| Need to strain when urinating | 0 | 1 | | 2 | | 3 |
| Feeling of incomplete emptying of the bladder after urination | 0 | 1 | | 2 | | 3 |
| Pain in the bladder | 0 | 1 | | 2 | | 3 |
| Pain in the urethra | 0 | 1 | | 2 | | 3 |
| CLSS (Sum of questions) ______ | | |  | |  | |
