# Supplementary material for: New combined surgery for cervical cancer complicated by pelvic organ prolapse using autologous fascia lata: A case report
Source: Clin Case Rep. 2020 May 20;8(8):1382–6. doi: 10.1002/ccr3.2883 (PMC7455420; doi:10.1002/ccr3.2883)
Supplement: Supplementary file 2 — Table S2 [file CCR3-8-1382-s002.docx]

Supplementary Table 2. Overactive Bladder Symptom Score Questionnaire (modified from Homma Y et al^7^)

Overactive bladder symptom score

| *Question* | *Frequency* | *Score* |
| --- | --- | --- |
| How many times do you typically urinate from waking in the morning until sleeping at night? | ≤7  8-14  ≥15 | 0  1  2 |
| How many times do you typically wake up to urinate from sleeping at night until waking in the morning? | 0  1  2 | 0  1  2 |
|  | ≥3 | 3 |
| How often do you have a sudden desire to urinate, which is difficult to defer? | Not at all  Less than once a week  Once a week or more  About once a day  2–4 times a day  5 times a day or more | 0  1  2  3  4  5 |
| How often do you leak urine because you cannot defer the sudden desire to urinate? | Not at all  Less than once a week | 0  1 |
|  | Once a week or more | 2 |
|  | About once a day | 3 |
|  | 2–4 times a day | 4 |
|  | 5 times a day or more | 5 |
